# Nurses’ Perspectives on the Non-Pharmacological Management of Oral Mucositis in Onco-Hematological Care: A Qualitative Content Analysis

**DOI:** 10.3390/nursrep16030100

**Published:** 2026-03-17

**Authors:** Orejeta Diamanti, Giovanna Artioli, Paolo Pellegrino, Francesca Bonadies, Matteo Bernardi, Alberto Camuccio, Mirsad Pasalic, Donato Antonio Rotondo, Federica Dellafiore

**Affiliations:** 1 Scientific Direction, Veneto Institute of Oncology IOV-IRCCS, 35128 Padua, Italy; 2 Department of Life Health Sciences and Health Professions, Link Unicampus University, 00165 Rome, Italy; g.artioli@unilink.it (G.A.); f.dellafiore@unilink.it (F.D.); 3 Azienda ULSS 2 Marca Trevigiana–Ospedale di Conegliano, 31100 Conegliano, Italy; paolo.pellegrino@aulss2.veneto.it; 4 Healthcare Professions, Veneto Institute of Oncology IOV-IRCCS, 35128 Padua, Italy; francesca.bonadies@iov.veneto.it (F.B.); matteo.bernardi@iov.veneto.it (M.B.); 5 Immunology and Molecular Oncology Diagnostics Unit, Veneto Institute of Oncology IOV-IRCCS, 35128 Padua, Italy; 6 Medical Inpatient Unit, Veneto Institute of Oncology IOV-IRCCS, 35128 Padua, Italy; mirsad.pasalic@iov.veneto.it; 7 Nursing and Public Health Sciences, University of Rome “Tor Vergata”, 00133 Rome, Italy; donato.rotondo@gmail.com

**Keywords:** nurses, onco-hematology, non-pharmacological treatment, oral mucositis management, supportive care, qualitative study

## Abstract

**Background/Objectives**: Oral mucositis (OM) is a common complication in onco-hematological patients undergoing chemotherapy and hematopoietic stem cell transplantation, negatively affecting comfort, nutrition, and quality of life. Despite existing assessment tools and recommendations, OM management—particularly non-pharmacological approaches—remains inconsistent, and evidence on nurses’ perspectives and contextual factors is limited. This study explored nurses’ perceptions and experiences regarding non-pharmacological treatments for OM, including educational needs and barriers and facilitators to implementation in clinical practice. **Methods**: A qualitative descriptive study using inductive content analysis was conducted. Semi-structured interviews were carried out with nurses working in onco-hematological settings in Italy. Data were analysed according to the Elo and Kyngäs framework. **Results**: Twelve nurses with extensive experience in onco-hematology and transplant care participated in the study. Five main themes emerged: (1) education and training pathways; (2) approaches to mucositis management; (3) nursing competence in OM care; (4) interprofessional collaboration; and (5) governance of practice, including protocols and guidelines. Findings highlighted strong experiential competence, high levels of nursing autonomy in assessment and patient education, and effective interprofessional collaboration, particularly in specialised settings. However, training pathways were predominantly informal, and the availability and use of protocols varied widely across clinical contexts. **Conclusions**: Non-pharmacological management of OM appears to be sustained primarily by advanced nursing competence and a specialised clinical culture rather than by structured education and standardised governance. Addressing educational gaps and promoting shared protocols may enhance the consistency, quality, and equity of supportive care while ensuring that the findings are clearly reflective of nurses’ experiences.

## 1. Introduction

Oral mucositis (OM) is a common and clinically significant complication in onco-hematologic patients undergoing chemotherapy and hematopoietic stem cell transplantation (HSCT) [1]. Characterised by inflammatory and ulcerative lesions of the oral mucosa, OM can severely compromise patients’ comfort, nutritional intake, and overall quality of life, representing a major challenge in daily clinical care. Its incidence varies according to treatment intensity, affecting approximately 30–40% of patients receiving standard-dose chemotherapy and rising to over 75% in those treated with high-dose regimens [2,3]. The highest rates are observed in HSCT settings, particularly following conditioning regimens, with OM occurring in up to 75–98% of allogeneic transplant recipients [4]. High prevalence has also been reported among patients with acute leukemia receiving induction chemotherapy and among individuals undergoing intensive treatment for multiple myeloma, underscoring the clinical burden of OM across different onco-hematologic populations [3,5,6].

Oral mucositis (OM) results from epithelial injury induced by cytotoxic agents, followed by inflammatory amplification and mucosal breakdown. Histopathological changes extend beyond the epithelial layer, involving the underlying connective tissue, vascular endothelium, and extracellular matrix. This disruption of mucosal integrity compromises the oral barrier and promotes microbial and translocation, thereby increasing susceptibility to local and systemic infections, including bacteraemia and sepsis, particularly in immunocompromised patients [7].

Given its high prevalence and serious clinical consequences and economic burden—including pain, impaired nutrition, increased risk of infection, diminished quality of life (QoL), higher healthcare costs, and extended hospitalization—the prevention and management of OM are critical components of supportive care in onco-hematology.

To support systematic assessment, several validated tools have been developed to grade OM severity, typically evaluating clinical signs, symptoms, pain intensity, and functional impairments such as swallowing difficulties using 4- or 5-point scales. A recent Delphi study identified the WHO Oral Toxicity Scale as the most widely used instrument, followed by the National Cancer Institute Common Terminology Criteria for Adverse Events (NCI-CTCAE) and the Oral Mucositis Assessment Scale (OMAS) [8].

Although nurses are primarily responsible for the assessment and management of oral mucositis, including daily oral assessment, preventive interventions, and patient education [9,10,11], oral care remains a frequently missed component of nursing care in hospitalized oncology patients, potentially contributing to poorer mucositis outcomes [12]. The integration of validated assessment tools into routine practice facilitates early identification of OM and timely intervention, potentially reducing morbidity and improving patient outcomes. Despite oral care being widely recognized as a priority in oncology nursing [13,14], evidence regarding actual clinical practices remains limited and fragmented [15,16]. Previous studies have documented gaps in nurses’ knowledge and inconsistencies in the application of evidence-based protocols [10,17]. Moreover, although clinical guidelines emphasize comprehensive OM care—including risk assessment, systematic oral examination, and patient and caregiver education—only a minority of patients report receiving nurse-led guidance on oral self-care practices [18,19].

Taken together, these findings highlight a persistent gap between available evidence-based recommendations and their translation into routine clinical practice. While validated assessment tools and non-pharmacological interventions for OM management are well described in the literature, their adoption and consistent use appear to vary considerably across clinical settings. This variability suggests that challenges extend beyond the availability of evidence and protocols, encompassing healthcare providers’ knowledge, experiential learning, educational preparation, and contextual factors influencing clinical decision-making.

To date, limited attention has been paid to exploring healthcare providers’ perceptions and experiences regarding the use of non-pharmacological treatments for oral mucositis in onco-hematological care. In particular, little is known about the educational and training needs perceived by healthcare providers, or about the facilitators and barriers that influence the implementation of these interventions in everyday clinical practice. In the Italian healthcare context, where supportive care for onco-hematological patients is delivered across heterogeneous clinical settings, evidence on these aspects remains particularly scarce. A deeper understanding of these experiential and contextual dimensions is essential to inform the development of targeted educational strategies and implementation approaches aimed at optimizing supportive care for patients at risk of or affected by oral mucositis.

Accordingly, the aim of this study was to explore nurses’ perceptions and experiences regarding the use of non-pharmacological treatments for oral mucositis in onco-hematological patients within the Italian healthcare setting. Additionally, the study sought to identify perceived educational and training needs, as well as facilitators and barriers influencing the implementation of these interventions in routine clinical practice.

## 2. Materials and Methods

### 2.1. Study Design

A qualitative descriptive study employing inductive content analysis was conducted to explore nurses’ perceptions and experiences regarding the use of non-pharmacological treatments for OM in onco-hematological patients. [20]. The reporting quality of the study was assessed using the Consolidated Criteria for Reporting Qualitative Research (COREQ) 32-item checklist [21]. The COREQ evaluation was performed independently by two studies (AC and PP), who reviewed the manuscript against each of 32 items to assess completeness and transparency of reporting. Each item was rated, and specific page/section references were documented (Supplementary File). Any discrepancies between reviewers were discussed until consensus was reached; when necessary, a third researcher (OD) was consulted to resolve remaining disagreements. Given the limited empirical knowledge regarding healthcare providers’ experiential perspectives, educational needs, and contextual factors influencing the use of non-pharmacological interventions for oral mucositis, an inductive approach was adopted. This approach allowed categories to emerge from participants’ accounts without imposing predefined theoretical frameworks, thereby capturing the complexity of real-world clinical practice [22,23].

### 2.2. Setting and Participants

The study was conducted in onco-hematological clinical settings in Italy, including oncology and hematology units, hematopoietic stem cell transplantation services, palliative and supportive care units, and intensive or emergency care areas dedicated to onco-hematological patients. A purposive sampling strategy was used to recruit nurses with direct clinical experience in the management of oral mucositis. Eligible participants were nurses involved in the care of onco-hematological patients—namely physicians (oncologists, hematologists, palliative care specialists, and hospital dentists), nurses, physiotherapists or oral rehabilitation therapists, and dental hygienists—who had at least five years of professional experience with patients undergoing chemotherapy and/or hematopoietic stem cell transplantation. Participants were required to have direct experience in assisting patients with oral mucositis and/or in implementing pharmacological and non-pharmacological interventions for its management, to be currently working in relevant onco-hematological settings, and to be willing to take part in a semi-structured interview after providing written informed consent. Fluency in the language used for the interview (Italian or English) was also required.

In line with the qualitative descriptive design and inductive content analysis, the sample size was not determined a priori through data saturation [24]. Instead, recruitment was guided by the concept of informational power, whereby the adequacy of the sample depends on the richness, relevance, and depth of the data in relation to the study aim. According to this approach, sufficient informational power is achieved when the sample provides nuanced and diverse insights that are directly pertinent to the research questions, taking into account factors such as the specificity of the sample, the quality of the interview dialogue, and the analytical strategy employed, rather than the mere absence of new themes [25].

### 2.3. Data Collection

Data were collected through semi-structured individual interviews conducted by trained qualitative researchers. An interview topic guide was developed based on the study aim and research questions and informed by existing literature on oral mucositis management. The guide included open-ended questions exploring nurses’ perceptions, experiences, and practices related to non-pharmacological treatments for oral mucositis, as well as perceived educational needs and contextual facilitators and barriers to implementation. Interviews began with a broad introductory question aimed at eliciting participants’ general views on the use of non-pharmacological treatments in onco-hematological care. Subsequent questions explored four main thematic areas: (1) knowledge and experience with non-pharmacological treatments; (2) perceptions of effectiveness and applicability in clinical practice; (3) educational and training needs; and (4) facilitators and barriers influencing implementation. Each interview concluded with an open-ended question allowing participants to add further reflections. Interviews were audio-recorded with participants’ consent and transcribed verbatim for analysis. Sessions lasted approximately 35–70 min, providing adequate depth in exploring participants’ experiences while maintaining consistency across data collection.

### 2.4. Sociodemographic and Professional Data

To characterise the study sample, sociodemographic, professional, and organisational data were collected, including age group, gender, professional role, years of professional experience, years of experience in caring for onco-hematological patients with oral mucositis, clinical setting, level of education, and previous training on non-pharmacological treatments for oral mucositis. Additional information regarding frequency of care for patients with oral mucositis, personal use of non-pharmacological treatments, access to clinical guidelines or protocols, type of healthcare institution, and geographical area (Northern, Central, Southern Italy, and Islands), was also collected.

### 2.5. Data Analysis

Data were analysed using inductive qualitative content analysis following the methodological framework proposed by Elo and Kyngäs [20,26]. The analytical process comprised three main phases: preparation, organising, and reporting [27]. During the preparation phase, interview transcripts were read repeatedly to achieve immersion and gain an overall understanding of the data. Meaning units relevant to the study aim were identified and open codes were generated. In the organising phase, codes with similar content were grouped into subcategories, which were then abstracted into broader categories reflecting shared meanings and patterns across participants’ accounts. Categories were continuously compared and refined to ensure internal consistency and conceptual clarity [26]. Two researchers independently conducted the coding and categorisation process. Discrepancies were discussed and resolved through consensus, with the involvement of a third researcher when necessary. Throughout the analysis, the research team engaged in iterative discussions and reflexive dialogue to ensure that findings remained grounded in the data. All stages of the analysis were conducted manually, in line with the scope of the study and the researchers’ methodological expertise [28].

### 2.6. Trustworthiness

Trustworthiness was ensured in accordance with established qualitative criteria, including credibility, dependability, confirmability, and transferability [29]. Credibility was enhanced through researcher triangulation, independent coding, and iterative engagement with the data. Dependability was supported by maintaining a transparent audit trail documenting analytical decisions. Confirmability was addressed through reflexive discussions among researchers and systematic linkage between data excerpts and emerging categories. Transferability was facilitated by providing detailed descriptions of the study context, participants, and analytical procedures. Reporting followed established qualitative reporting guidelines to enhance transparency and rigor [20].

Participant validation (member checking) was not performed, as the qualitative descriptive design aimed to capture shared experiential patterns rather than individual narrative reconstruction. Credibility was instead strengthened through researcher triangulation, iterative data analysis, reflexive discussions, and maintenance of detailed audit trail, consistent with methodological recommendations for qualitative content analysis.

### 2.7. Ethical Consideration

Ethical approval for the study was obtained from the Regional Ethics Committee of the Azienda Ospedaliera of Perugia, Umbria, Italy (protocol number 2028/2025, approved on 14 April 2025). All procedures were conducted in accordance with the Declaration of Helsinki. Participants received detailed information about the study aims, procedures, confidentiality measures, and their right to withdraw at any time without consequences. Written informed consent was obtained prior to data collection. Anonymity and data protection were ensured through the use of unique identification codes, and findings are reported in aggregated form to prevent participant identification.

## 3. Results

### 3.1. Sample Characteristics

A total of 14 nurses were interviewed; of these, 12 interviews were included in the final analysis, while 2 were excluded as they were conducted as pilot interviews to test and refine the interview guide. The final sample comprised 12 nurses, the majority of whom were female (*n* = 9), with a mean age of 44.8 years. Participants reported extensive professional experience, with a mean of 21 years as registered nurses and an average of 16.3 years working specifically in onco-haematology and haematopoietic stem cell transplantation settings. At the time of the study, five participants were employed in transplant centres, four in haematology units, and three in onco-haematology wards. Overall, the sample reflected a highly experienced group of nurses with long-standing involvement in the care of patients affected by oral mucositis. A detailed description of participants’ socio-demographic and professional characteristics is provided in Table 1.

### 3.2. Overview of Content Analysis Findings

The inductive content analysis generated a total of 47 codes, reflecting the breadth and complexity of nurses’ experiences in managing oral mucositis in onco-haematological settings. The most frequently occurring codes were Evidence, Mucositis assessment, and Patient education, highlighting the centrality of evidence-informed practice, systematic oral evaluation, and educational activities in participants’ accounts (Table S1—Supplementary File).

Through the process of abstraction and categorisation, five overarching themes were identified: (1) Education and competence; (2) Mucositis management; (3) Nursing Competence in Mucositis Management; (4) Interprofessional Collaboration in Mucositis Management; and (5) Governance of Practice—Protocols and Guidelines. Together, these themes describe nurses’ experiential knowledge, clinical practices, professional roles, and organisational contexts related to the use of non-pharmacological approaches for oral mucositis management. An overview of themes and sub-themes is presented in Table 2.

### 3.3. Theme 1—Education and Competence

Theme 1 focuses on how nurses acquire and develop the competencies required for managing oral mucositis, highlighting the educational pathways, training opportunities and professional culture that form the foundation of their specialised expertise, which supports autonomous practice explored in Theme 3. This theme is therefore centred on nurses’ formal and informal training experiences rather than their application of autonomy in clinical decision-making. Three sub-themes emerged: (1) personal education and specialisation, (2) continuing and (3) organisational training, and professional culture.

*1.1 Personal Education and Specialisation*. Most participants reported long-standing experience in onco-haematology and transplant units, often developed through progressive professional pathways combining individual training initiatives and extensive clinical practice. These quotes illustrate the acquisition of specialised knowledge and skills rather than autonomous decision-making Nurses consistently emphasised the highly specialised nature of onco-haematological care and the need for dedicated competencies. “I’ve been working in onco-haematology for almost twenty years… I believe in specialisation.” (P02). The following quote highlights the perceived gap in formal undergraduate education. “At university, we only touched on mucositis, but without practical examples.” (P04)

*1.2 Continuing and Organisational Training*. Although oral mucositis was described as a major care priority, nurses frequently reported limited access to structured training on its management. Dedicated organisational courses were often lacking, and learning was commonly described as informal or experience-based.

“Since I started working, no dedicated course: I learn from university and from colleagues.” (P03). This illustrates experiential learning as a supplement to formal education.

On-the-job shadowing with more experienced colleagues emerged as the most common training strategy, while a minority of participants described contexts characterised by well-structured, continuous, and multidisciplinary educational programmes.

“We review procedures every two years, and we used to have lessons with doctors on new therapies and guidelines.” (P11)

*1.3 Professional Culture.* Participants highlighted the importance of a shared, specialised professional culture in onco-haematology, particularly in relation to standardisation of care practices. Several nurses stressed that this field requires dedicated expertise and should be delivered in appropriately specialised settings. There was a feeling also of a common professional culture between haematological nurses.

“Onco-haematology should only be practised in dedicated centres.” (P02)

In addition, participants expressed the need for greater uniformity in clinical behaviours and for the adoption of shared best practices, not only in relation to oral mucositis management but across the broader onco-haematological care pathway. These quotes reflect the cultural and organisational context of education and training, setting the stage for discussion of autonomy in Theme 3. “We need standard behaviours, shared best practices.” (P011)

### 3.4. Theme 2—Mucositis Management

Theme 2 describes nurses’ experiences related to the clinical management of oral mucositis in onco-haematology and transplant settings. Three sub-themes were identified: (1) evolution and incidence, (2) assessment and monitoring, and (3) prevention and treatment.

*2.1 Evolution and incidence*. Participants consistently reported oral mucositis as a highly prevalent condition, particularly among patients undergoing haematopoietic stem cell transplantation. While mucositis was described as almost universal in this population, several nurses perceived a reduction in severity compared to earlier stages of their professional experience.

“I don’t recall any transplant patient who didn’t develop at least grade 1 mucositis.” (P07)

“Severe mucositis is now much rarer than it was years ago.” (P02)

*2.2 Assessment and monitoring*. Oral cavity assessment was described as a core nursing activity and considered an essential component of daily care. In transplant settings, assessment was reported as systematic and frequent, whereas in other wards practices varied in terms of frequency and formalisation.

“For me, assessing the oral cavity is part of checking vital signs.” (P02)

“In transplant units, we assess the mouth every day.” (P07)

The use of validated assessment scales was widespread but inconsistent, with different wards adopting different tools or locally developed scales. Documentation practices were also described as heterogeneous.

“We use the WHO scale.” (P05)

“It’s a scale created by the ward, not a standard one.” (P03)

*2.3 Prevention and treatment*. Preventive practices were primarily based on oral hygiene education and the use of mouthwashes, including chlorhexidine, benzydamine hydrochloride, and sodium bicarbonate. These interventions were generally initiated at admission and reinforced through patient education.

“As soon as they’re admitted, we give them chlorhexidine rinses.” (P02)

Once mucositis developed, treatment followed a stepwise approach, starting with local measures and progressing to pain management and nutritional support in more severe cases. The use of complementary non-pharmacological interventions was limited and largely confined to cryotherapy, he use of ice pops in practice, and mouthwashes.

“Ice lollies help relieve the pain a bit.” (P03)

### 3.5. Theme 3—Nursing Competence in Mucositis Management

While Theme 1 focused on how nurses acquired and developed their competencies through educational pathways, training, and the consolidation of a shared professional culture, Theme 3 shifts the focus to how these acquired competencies are applied in everyday clinical practice, reflecting nurses’ autonomy, decision-making, and professional responsibility. This theme therefore emphasises the practical use of competence rather than its acquisition. It explores nurses’ perceptions of the professional role, autonomy, and responsibilities they exercise as a result of their specialised expertise in mucositis management. These dimensions of professional practice are articulated across three interrelated areas: (1) specialised clinical culture, (2) clinical autonomy and responsibility, and (3) patient education.

*3.1 Specialised Clinical Culture.* All participants described a strong sense of belonging to a highly specialised clinical environment, characterised by shared experiential knowledge and a commitment to evidence-based practice. Onco-haematology was perceived as a field requiring specific expertise, not easily transferable to other clinical contexts, and as a clinical care setting where specialised competence is actively applied in everyday clinical decision-making.

“Onco-haematology is not for everyone.” (P01)

Several nurses highlighted how this specialised culture legitimises advanced nursing practice and reinforces high clinical standards, enabling them to apply their specialised expertise with confidence.

“In onco-haematology, you can’t afford to work without basing your practice on evidence.” (P01)

“There was obsessive attention… haematology was taken very seriously, both in transplants and in the ordinary ward.” (P02)

*3.2 Clinical Autonomy and Responsibility*. Within this shared culture, nurses reported a high level of autonomy and clinical responsibility in the prevention, assessment, and early management of mucositis. These examples illustrate the practical exercise of competence, distinct from the formal training described in Theme 1. Nurses emphasised that this autonomy derives directly from the specialised competences developed through dedicated training pathways and, above all, refined over years of practice, and is exercised as an integral part of their professional role. Oral cavity assessment was described as a core nursing competence, integrated into routine clinical evaluation.

“A nurse in onco-haematology who doesn’t look in a patient’s mouth… for me, they should work elsewhere.” (P02)

Nurses frequently positioned themselves as the first professionals to detect early signs of mucositis, such as pain or difficulty eating, and to initiate communication with physicians.

“Usually, we’re the first to notice that something is wrong.” (P03)

“The whole grading and scoring system… that’s done by nurses. It remains their evaluation.” (P012)

*3.3 Patient Education as a Core Nursing Competence*. Patient education emerged as a central and autonomous nursing competence, delivered across the entire care trajectory, including pre-admission and hospitalisation phases. This theme highlights the application of knowledge in supporting patients, rather than the acquisition of skills. Educational interventions focused on symptom prevention, self-care behaviours, and expectation management. Participants described education not as a simple informational task, but as a core ex-pression of their clinical and care-related competence.

“We explain to the patient what to expect, when mucositis might appear, and how to behave.” (P02)

Beyond its practical value, education was also recognised as a key strategy for emotional support and reassurance. It illustrates the integration of specialised knowledge, autonomous practice, and therapeutic communication. It also plays an important role in supporting patients, particularly when they experience pain the onset of mucositis. This occurs not only by preparing patients for when to expect it—such as during pre-admission or admission-day education—but also by offering precise, timely information and management advice at the moment of need. In doing so, nurses draw on their specialised knowledge to support both symptom management and the therapeutic relationship. Thus, practical guidance and emotional support are closely intertwined, reflecting autonomous professional practice.

“When we see them frightened by the pain, we try to reassure them and explain that it’s temporary.” (P03)

### 3.6. Theme 4—Interprofessional Collaboration in Mucositis Management

Theme 4 explores nurses’ experiences of interprofessional collaboration in mucositis management, highlighting two interrelated dimensions: (1) professional trust and (2) multidisciplinary integration.

*4.1 Professional Trust.* Collaboration between nurses and physicians was described as a core feature of onco-haematology and transplant settings, grounded in mutual trust and recognition of nursing clinical expertise. Nurses reported that medical decision-making frequently relied on shared or nurse-initiated assessments, particularly in the monitoring of oral cavity conditions.

“We trust our own evaluations, and we coordinate with the doctor when there’s a worsening.” (P04)

In some contexts, this trust was further reinforced through shared educational experiences and joint updates on therapies and guidelines.

“We used to have lessons with doctors on new therapies and guidelines, both medical and nursing.” (P011)

These dynamics positioned nurses as active clinical partners rather than passive executors of medical prescriptions.

*4.2 Multidisciplinary Integration.* Beyond the doctor–nurse dyad, mucositis management was described as a collective process involving dietitians, pharmacists, and healthcare assistants, each contributing distinct but integrated competencies. Collaboration with dietitians was portrayed as particularly interactive and problem-oriented.

“We consult the dietitian, especially when the patient can’t eat: we decide together what to suggest.” (P03)

Interactions with pharmacists were more structured and mediated by medical prescriptions; however, nurses with advanced or consultancy roles described greater involvement in customised solutions.

“Together with the compounding pharmacy, I get customised mouthwashes made.” (P012)

Healthcare assistants were recognised as playing a meaningful supportive role, particularly in patient motivation and daily care, although nurses remained cautious about delegating oral hygiene due to the clinical complexity of mucositis.

“They help the patient with oral hygiene only if guided by us.” (P06)

### 3.7. Theme 5—Governance of Practice—Protocols and Guidelines

Theme 5 addresses the organisational frameworks guiding mucositis management, revealing marked variability in the availability and use of protocols and guidelines. Two dimensions emerged: (1) coexistence of discretionary and standardised practices, and a (2) strong demand for standardisation.

*5.1 Discretion versus Standardisation*. Participants described heterogeneous organisational scenarios. In highly specialised centres, particularly transplant units, mucositis management was supported by regularly updated protocols and institutional guidelines.

“Procedures are reviewed every two years… including those for mucositis and oral cavity management.” (P011)

In contrast, other settings lacked formal references, relying predominantly on individual experience and locally adapted practices.

“We don’t have a standard or a procedure: we rely more on experience.” (P07)

Several wards occupied an intermediate position, using outdated or informally adapted protocols, which nurses perceived as insufficient to guide complex clinical decisions.

“There are protocols, but they’re poorly updated. We rely heavily on personal experience.” (P08)

A recurrent concern across settings was the absence of clear guidance on assessment frequency, documentation, and oral pain management.

“We need a specific protocol defining checks and parameters to record in the chart.” (P06)

*5.2 Demand for Standardisation.* In response to this fragmentation, nurses consistently expressed the need for harmonised, shared, and updated protocols, ideally applicable across onco-haematology settings.

“My dream is to achieve uniform behavior… shared best practices.” (P02)

Standardisation was seen not as a constraint but as a means to support clinical decision-making, ensure continuity of care, and reinforce professional competence.

“A protocol with precise steps: prevention, constant monitoring…” (P10)

## 4. Discussion

This study provides an in-depth exploration of nurses’ perceptions and experiences regarding the use of non-pharmacological treatments for oral mucositis in onco-haematological settings, highlighting a complex interplay between professional competence, education, and organisational frameworks. The findings offer a critical understanding of how non-pharmacological mucositis management is enacted in everyday clinical practice, showing that care relies primarily on advanced nursing competence, experiential knowledge, and interprofessional trust rather than on formalised education or standardised governance. This study adds to the existing literature by shifting the focus from efficacy of interventions to the experiential, organisational, and professional conditions that shape their real-world implementation—an area that remains underexplored despite the high clinical burden of oral mucositis [30].

From a practice perspective, several evidence-based strategies may support improvement in OM management, including implementation of structured oral care protocols, systematic use of validated assessment tools, multidisciplinary education programmes, and nurse-led patient education interventions [3]. Previous studies have demonstrated that standardized oral care bundles and interprofessional training initiatives can reduce variability in care delivery and improve patient outcomes [31]. Addressing missed nursing care—particularly oral hygiene support—represents an additional priority, as inadequate preventive care may contribute to grater mucositis severity and increase healthcare burden [16].

### 4.1. Education and Competence in Oral Mucositis Management

Participants consistently described oral mucositis as a central and highly prevalent clinical issue, particularly in haematopoietic stem cell transplantation settings, underlining its relevance as a nursing-sensitive condition that demands proactive monitoring and management [32]. Despite this centrality, nurses perceived undergraduate nursing education as offering limited preparation for the practical prevention and management of mucositis. This gap appears to be filled primarily through experiential learning, informal peer support, and on-the-job training, rather than through structured educational pathways.

These findings are consistent with previous literature indicating that complex supportive care needs in oncology are often insufficiently addressed within pre-licensure nursing curricula, leaving nurses to rely heavily on experiential and context-based learning once in practice [33]. While such learning trajectories can foster deep clinical expertise, they may also contribute to heterogeneity in practice and uneven skill development across settings. In the present study, continuing and organisational training on oral mucositis was reported as sporadic and context-dependent, echoing concerns raised in prior studies on oncology nursing education and continuing professional development [34,35].

Our findings highlight the tension between experiential expertise and the lack of standardisation. While such learning trajectories foster advanced clinical competence, they also contribute to variability in practice and uneven skill development across settings. Continuing and organisational training was reported as sporadic and context-dependent, reflecting similar challenges described in oncology nursing education and professional development.

The study revealed that the use of non-pharmacological interventions beyond cryotherapy and mouthwashes was limited. This low adoption may reflect both organisational and governance barriers, such as variability in the availability and use of protocols, and perceived gaps in formal training, which lead nurses to rely primarily on experiential knowledge. These contextual factors highlight the complex interplay between professional competence, education, and institutional frameworks in shaping OM management practices, and suggest areas for improvement in both structured training and guideline implementation.

### 4.2. Tacit Knowledge and the Challenge of Standardisation

One of the most salient and paradoxical findings of this study is the coexistence of a highly developed shared professional culture with a fragmented level of formal standardisation in oral mucositis management. Nurses described strong experiential knowledge, refined clinical judgement, and a shared understanding of effective practices, often in the absence of updated, harmonised protocols or institutional guidelines. This demonstrates that much of nursing practice in this context is guided by tacit knowledge rather than codified standards [36]. To improve consistency and quality of care, structured practices such as standardised oral care protocols, routine assessment using established tools, patient education, and interdisciplinary training should be prioritised. Formalising these approaches allows tacit expertise to be translated into reliable and replicable practice, ensuring high-quality care across settings. Integrating experiential knowledge with structured procedures enhances both the effectiveness and equity of mucositis management.

Tacit knowledge—experiential, context-dependent, and often unarticulated—remains a cornerstone of advanced nursing practice in complex clinical environments [33]. However, when tacit knowledge is not explicitly linked to organisational standards, variability in care may occur, highlighting the importance of translating individual expertise into shared practices. Nurses perceived the absence of clear guidance regarding assessment, documentation, and treatment pathways as a critical gap, underscoring the need for deliberate strategies to integrate tacit and explicit knowledge into standardised care [37].

Importantly, participants did not perceive standardisation as bureaucratic; rather, they saw it as a supportive framework that reinforces professional competence, ensures continuity of care, and legitimises clinical judgement. Nurses in specialised centres described participatory models for developing and revising protocols, illustrating how experiential knowledge can be formally recognised and incorporated into standardised pathways [37].

The average experience of the participants was 21 years, which may have influenced the prominence of tacit knowledge in practice. Highly experienced nurses likely rely more on accumulated experiential expertise and refined clinical judgment, potentially reducing their dependence on formal protocols. It is possible that nurses with less experience might demonstrate a different balance between tacit knowledge and formal guidelines, relying more heavily on structured protocols and organisational support. These considerations highlight the contextual nature of our findings and the potential variability in practice across nurses with differing levels of experience.

Additionally, our findings suggest that participatory governance models, where nurses are actively involved in translating their experiential knowledge into shared and standardised protocols, may help reduce variability in OM management. By formalising best practices derived from clinical experience, such models can support consistent, high-quality care across different settings while reinforcing professional accountability and ownership of practice. Integrating experiential expertise into structured guidelines ensures that tacit knowledge contributes to sustainable organisational memory and equitable patient care.

### 4.3. Professional Culture and Interprofessional Collaboration

The findings further underscore the role of a strong professional culture in sustaining high standards of care in onco-haematology. Nurses articulated a clear sense of belonging to a specialised clinical community, characterised by shared values, evidence-informed practice, and high expectations regarding clinical competence. Such professional cultures have been described as critical enablers of advanced nursing practice and professional autonomy in specialised care settings, where specialist nurses contribute significantly to the development of patient safety culture and organisational quality initiatives [38].

As recognized in other settings [39,40], interprofessional collaboration was identified as a key facilitator in mucositis management. Trust-based relationships with physicians enabled nurses to exercise clinical autonomy within shared decision-making processes. Ongoing dialogue and joint educational activities reinforced learning and professional recognition [41]. Beyond physicians, collaboration with dietitians, pharmacists, and healthcare assistants supported a holistic approach to patient care. Despite this, nurses remained cautious in delegating oral care tasks due to the clinical complexity of mucositis, emphasising the need for specialised competence [42].

### 4.4. Limitations

This study has several limitations. First, the sample included only nurses, limiting transferability to other professional groups involved in mucositis management. Second, the study was conducted within the Italian healthcare system, which may differ from other contexts. Third, participants were highly experienced, possibly accentuating the emphasis on tacit knowledge and autonomy. Finally, as with all qualitative research, findings are context-specific and aim to provide in-depth understanding rather than generalisability. Nevertheless, the rich descriptions support analytical transferability to similar clinical and organisational contexts.

### 4.5. Implications for Education and Practice

Taken together, these findings highlight the need for a more coherent integration of oral mucositis management within nursing education and continuing professional development. At the undergraduate level, greater emphasis should be placed on supportive care, symptom management, and applied clinical reasoning for oncology-specific complications.

At the organisational level, structured and continuous training programmes—delivered through multidisciplinary approaches—can reduce over-reliance on informal learning. Participatory models for guideline development involving nurses can translate experiential knowledge into shared standards. Such strategies enhance consistency and quality of care, reinforce professional identity and accountability, and foster organisational learning in complex care settings.

Importantly, these approaches are likely to improve patient outcomes by reducing mucositis severity, complications, and treatment interruptions, while also optimising resource use and reducing costs associated with extended hospital stays or additional interventions.

## 5. Conclusions

This study highlights that the non-pharmacological management of oral mucositis in onco-hematological settings is largely sustained by advanced nursing competence, experiential knowledge, and strong interprofessional collaboration, rather than by formalised educational pathways and standardised organisational frameworks. Nurses emerge as key clinical actors in the assessment, early detection, and patient education related to oral mucositis, yet their practice is often shaped by local culture and individual experience rather than shared protocols. The marked variability observed across settings underscores the need to move from an experience-dependent model toward structured, competence-based education and harmonised clinical guidelines. Strengthening educational preparation, supporting interprofessional training, and promoting consistent governance of practice may enhance the quality, safety, and equity of supportive care for patients at risk of or affected by oral mucositis in onco-hematology.

## Figures and Tables

**Table 1:** Sociodemographic characteristics of sample (12 nurses).

ID	Age (Years)	Sex	Professional Experience (Years)	Experience in Haematology (Years)	Current Ward/Unit	Highest Educational Qualification	Specific Training in Mucositis	Frequency of Care for Patients with Mucositis	Availability of Protocols/Guidelines	Geographical Area
P01	34	M	10	7	Transplant Centre and Haematology	MSc in Nursing; two postgraduate Master’s degrees; currently enrolled	No, only self-directed learning and ward-based training	High	Yes	Northern Italy
P02	45	M	20	20	Haematology	MSc in Nursing; MSc in Nutritional Sciences; postgraduate Master’s degree	No, only self-directed learning and ward-based training	High	No	Southern Italy
P03	29	F	5	5	Onco-haematology	Bachelor’s degree in Nursing	No, on-the-job training	High	No	North-East Italy
P04	44	F	10	9	Onco-haematology	Bachelor’s degree in Nursing	No, on-the-job training	High	No	North-East Italy
P05	45	F	23	18	Haematology	Bachelor’s degree in Nursing	No, on-the-job training	High	No	Central Italy
P06	43	F	20	20	Transplant Centre	Bachelor’s degree in Nursing	No, only self-directed learning and ward-based training	High	No	Central Italy
P07	46	F	22	22	Transplant Centre	Bachelor’s degree in Nursing	No, only on-the-job training	High	No	Central Italy
P08	55	F	34	34	Haematology	Bachelor’s degree in Nursing	Yes, online (distance learning) courses	High	Yes, but not updated	Northern Italy
P09	57	F	35	35	Transplant Centre	Bachelor’s degree in Nursing	Yes	High	Yes	North-East Italy
P10	55	F	35	4	Onco-haematology	Bachelor’s degree in Nursing	No	High	No	North-East Italy
P11	32	F	9	8	Onco-haematology/Transplant Centre	MSc in Nursing	Yes	High	Yes	North-East Italy
P12	53	M	30	20	Haematology	MSc in Nursing	Yes	High	Yes	Northern Italy

**Table 2:** Overview of themes and sub-themes derived from inductive content analysis.

Theme	Sub-Themes	Description/Focus
*Theme 1*. Education and Competence	1.1 Personal education and specialisation	Progressive development of expertise through long-term clinical experience in onco-haematology and transplant settings; undergraduate education perceived as insufficient for practical mucositis management.
	1.2 Continuing and organisational training	Limited availability of structured training on oral mucositis; reliance on informal, experience-based learning and peer support.
	1.3 Professional culture	Shared specialised professional culture emphasising expertise, standardisation of practices, and dedicated care settings.
*Theme 2*. Mucositis Management	2.1 Evolution and incidence	Oral mucositis perceived as highly prevalent, particularly in HSCT patients, with a perceived reduction in severity over time.
	2.2 Assessment and monitoring	Oral cavity assessment recognised as a core nursing activity; variability in frequency, documentation, and use of validated tools.
	2.3 Prevention and treatment	Preventive oral care, patient education, and mouthwashes as primary strategies; limited use of other non-pharmacological interventions.
*Theme 3*. Nursing Competence in Mucositis Management	3.1 Specialised clinical culture	Strong sense of belonging to a specialised environment legitimising advanced nursing practice and evidence-informed care.
	3.2 Clinical autonomy and responsibility	High level of nursing autonomy in assessment, early detection, and management of mucositis.
	3.3 Patient education as a core competence	Patient education as a central nursing role encompassing prevention, self-care, and emotional support.
*Theme 4*. Interprofessional Collaboration in Mucositis Management	4.1 Professional trust	Trust-based nurse–physician collaboration supporting shared decision-making and recognition of nursing expertise.
	4.2 Multidisciplinary integration	Collaboration with dietitians, pharmacists, and healthcare assistants within an integrated care approach.
*Theme 5*. Governance of Practice—Protocols and Guidelines	5.1 Discretion versus standardisation	Coexistence of formal protocols, outdated guidelines, and discretionary practices leading to variability in care.
	5.2 Demand for standardisation	Strong demand for harmonised, updated, and shared protocols to support clinical decision-making and continuity of care.

## Data Availability

The original contributions presented in this study are included in the article/Supplementary Material. Further inquiries can be directed to the corresponding authors.

## Supplementary Materials

The following supporting information can be downloaded at: https://www.mdpi.com/article/10.3390/nursrep16030100/s1, Consolidated criteria for reporting qualitative studies (COREQ): 32-item checklist; Table S1: The frequence of differences codes emerged by analysis.

## Public Involvement Statement

Only nurses were involved in the research.

## Guidelines and Standards Statement

This qualitative research manuscript was drafted against the Consolidated criteria for reporting qualitative studies (COREQ), of Tong A, Sainsbury P, Craig J. Consolidated criteria for reporting qualitative research (COREQ): a 32-item checklist for interviews and focus groups. International Journal for Quality in Health Care. 2007. Volume 19, Number 6: pp. 349–357.

## Abbreviations

The following abbreviations are used in this manuscript:

OM	Oral Mucositis
COREQ	Consolidated Criteria for Reporting Qualitative Studies
NCI-CTCAE	Terminology Criteria for Adverse Events
WHO	World Health Organization
OMAS	Oral Mucositis Assessment Scale
QoL	Quality of Life
