# Supplementary material for: Nurses’ Perspectives on the Non-Pharmacological Management of Oral Mucositis in Onco-Hematological Care: A Qualitative Content Analysis
Source: Nurs Rep. 2026 Mar 17;16(3):100. doi: 10.3390/nursrep16030100 (PMC13029019; doi:10.3390/nursrep16030100)
Supplement: Supplementary file 1 [file nursrep-16-00100-s001.zip › nursrep-4161354-supplementary.pdf]

**Supplementary file**

# Consolidated criteria for reporting qualitative studies (**COREQ**): 32-item checklist

| Item No | Description | Reported on Page # |
|---|---|---|
| Domain 1: Research team and reflexivity | | |
| Personal Characteristics | | |
| 1. Interviewer/ facilitator | AC and PP conducted the interviews | Pg 3 |
| 2. Credentials | AC, RN, MSCN; PP, RN | N/A |
| 3. Occupation | Accademic nurse research; Nurse research | N/A |
| 4. Gender | | N/A |
| 5. Experience and training | Trained researchers | Pg 4 |
| Relationship with participants | | |
| 6. Relationship established | No prior relationship | N/A |
| 7. Participant knowledge of the interviewer | Participants informed about study aims - Consent | Pg 4 |
| 8. Interviewer characteristics | Reflexive discussion within team reported | Pg 5 |
| Domain 2: study design | | |
| Theoretical framework | | |
| 9. Methodological orientation and Theory | Inductive qualitative content analysis | Pg 3-5 |
| Participant selection | | |
| 10. Sampling | Purposive sampling | Pg 5-6 |
| 11. Method of approach | Invitation through professional/clinical settings (mail and telephone, email) | Pg 3-5 |
| 12. Sample size | Reported in Results section (N=14 nurses) | Pg 5-6 |
| 13. Non-participation Setting | Two were excluded as per pilot interviews | Pg 5 |
| 14. Setting of data collection | Clinical work place settings | Pg 6 |
| 15. Presence of non participants | Not applicable | N/A |

| Item No | Description | Reported on Page # |
|---|---|---|
| 16. Description of sample | Demographic and professional characteristics reported | Pg 7 |
| **Data collection** | | |
| 17. Interview guide | Semi-structured guide described | Pg 4 |
| 18. Repeat interviews | No | N/A |
| 19. Audio/visual recording | Audio recorded | Pg 4 |
| 20. Field notes | Not reported | N/A |
| 21. Duration | N/A | N/A |
| 22. Data saturation | Discussed using informational power | Pg 4 |
| 23. Transcripts returned | No | N/A |
| **Domain 3: analysis and findings** | | |
| **Data analysis** | | |
| 24. Number of data coders | Two independent coders | Pg 4 |
| 25. Description of the coding tree | No | N/A |
| 26. Derivation of themes | Inductive derived from the data | Pg 8-9 |
| 27. Software | Manual analysis | Pg 5 |
| 28. Participant checking | No | N/A |
| **Reporting** | | |
| 29. Quotations presented | Yes, with participant ID | Pg 7 |
| 30. Data and findings consistent | Yes | Pg 6-13 |
| 31. Clarity of major themes | Yes | Pg9-13 |
| 32. Clarity of minor themes | Yes | Pg 9-13 |

Tong A, Sainsbury P, Craig J. *Consolidated criteria for reporting qualitative research (COREQ): a 32-item checklist for interviews and focus groups*. International Journal for Quality in Health Care. 2007. Volume 19, Number 6: pp. 349 – 357

**Table S1.** The frequence of differences codes emerged by analysis

| Code | Total frequency | P01 | P02 | P03 | P04 | P05 | P06 | P07 | P08 | P09 | P10 | P11 | P12 |
|---|---|---|---|---|---|---|---|---|---|---|---|---|---|
| Scientific evidence | 41 | 0 | 8 | 4 | 4 | 4 | 3 | 4 | 4 | 0 | 0 | 3 | 5 |
| Assessment of oral mucositis | 35 | 0 | 5 | 4 | 2 | 0 | 1 | 3 | 0 | 0 | 0 | 2 | 7 |
| Patient education | 30 | 4 | 2 | 5 | 2 | 1 | 1 | 2 | 0 | 0 | 2 | 2 | 2 |
| Prevention | 21 | 1 | 4 | 1 | 3 | 1 | 1 | 0 | 0 | 0 | 0 | 1 | 4 |
| Use of ice/ice pops (oral cryotherapy) | 21 | 1 | 1 | 1 | 2 | 2 | 4 | 1 | 0 | 0 | 0 | 0 | 1 |
| Professional experience | 18 | 1 | 0 | 0 | 1 | 2 | 3 | 2 | 0 | 0 | 2 | 4 | 0 |
| Interprofessional collaboration | 16 | 0 | 0 | 3 | 3 | 0 | 0 | 0 | 0 | 0 | 0 | 0 | 6 |
| Non-pharmacological interventions | 15 | 2 | 0 | 0 | 0 | 0 | 0 | 0 | 0 | 0 | 3 | 0 | 1 |
| Nursing responsibility | 15 | 2 | 4 | 2 | 2 | 0 | 1 | 1 | 0 | 0 | 0 | 1 | 2 |
| Nursing staff | 14 | 0 | 5 | 1 | 1 | 0 | 0 | 1 | 0 | 0 | 0 | 0 | 2 |
| Staff training | 14 | 0 | 0 | 0 | 0 | 0 | 4 | 2 | 0 | 0 | 1 | 4 | 0 |
| Progression of oral mucositis | 14 | 0 | 4 | 0 | 0 | 1 | 1 | 1 | 0 | 0 | 1 | 1 | 3 |
| Complementary therapies | 14 | 0 | 2 | 3 | 1 | 2 | 1 | 1 | 2 | 0 | 0 | 2 | 0 |
| Nursing autonomy | 12 | 0 | 0 | 2 | 1 | 1 | 2 | 3 | 0 | 0 | 0 | 1 | 0 |
| Relationships with other healthcare professionals | 12 | 3 | 0 | 0 | 0 | 3 | 3 | 0 | 0 | 0 | 0 | 2 | 0 |
| Pharmacological therapy | 10 | 1 | 2 | 0 | 0 | 0 | 1 | 0 | 0 | 0 | 0 | 1 | 2 |
| Education and training | 10 | 3 | 0 | 5 | 0 | 0 | 0 | 0 | 0 | 0 | 0 | 0 | 0 |
| Specific training | 10 | 0 | 7 | 0 | 0 | 0 | 0 | 0 | 0 | 0 | 0 | 0 | 2 |
| Protocols | 9 | 3 | 0 | 0 | 0 | 0 | 0 | 0 | 0 | 0 | 0 | 0 | 0 |
| Oral hygiene | 9 | 2 | 0 | 0 | 0 | 0 | 0 | 1 | 0 | 0 | 0 | 1 | 0 |
| Incidence and perception of oral mucositis | 8 | 0 | 0 | 0 | 0 | 1 | 1 | 1 | 0 | 0 | 0 | 1 | 0 |
| Assessment scales | 8 | 0 | 0 | 2 | 1 | 2 | 1 | 0 | 0 | 0 | 0 | 2 | 0 |
| Supportive care therapies | 7 | 0 | 1 | 2 | 4 | 0 | 0 | 0 | 0 | 0 | 0 | 0 | 0 |
| Pain | 6 | 0 | 0 | 1 | 1 | 0 | 0 | 0 | 0 | 0 | 1 | 0 | 0 |
| Nutrition | 6 | 1 | 1 | 0 | 0 | 1 | 0 | 0 | 0 | 0 | 0 | 1 | 0 |
| Management of oral mucositis | 6 | 0 | 0 | 3 | 0 | 0 | 1 | 1 | 0 | 0 | 0 | 1 | 0 |
| Organisation of care | 5 | 0 | 1 | 1 | 0 | 0 | 0 | 0 | 0 | 0 | 0 | 0 | 3 |
| Teamwork | 4 | 0 | 0 | 0 | 0 | 0 | 0 | 0 | 1 | 0 | 0 | 0 | 0 |

| | | | | | | | | | | | | | |
|---|---|---|---|---|---|---|---|---|---|---|---|---|---|
| Resources | 4 | 0 | 0 | 1 | 0 | 0 | 0 | 0 | 0 | 0 | 0 | 0 | 0 |
| Differences between haematology units and transplant centres | 4 | 0 | 3 | 0 | 0 | 0 | 0 | 0 | 0 | 0 | 0 | 1 | 0 |
| Staff turnover | 2 | 1 | 0 | 0 | 0 | 0 | 0 | 0 | 0 | 0 | 0 | 0 | 0 |
| Clinical documentation | 2 | 0 | 0 | 0 | 2 | 0 | 0 | 0 | 0 | 0 | 0 | 0 | 0 |
| Staff turnover | 2 | 0 | 1 | 0 | 0 | 0 | 0 | 0 | 0 | 0 | 0 | 1 | 0 |
| Therapeutic adjustment | 1 | 0 | 0 | 0 | 1 | 0 | 0 | 0 | 0 | 0 | 0 | 0 | 0 |
| Workplace-based learning | 1 | 0 | 0 | 0 | 1 | 0 | 0 | 0 | 0 | 0 | 0 | 0 | 0 |
| Routine nursing activities | 1 | 0 | 0 | 0 | 1 | 0 | 0 | 0 | 0 | 0 | 0 | 0 | 0 |
| Workload | 1 | 0 | 0 | 0 | 1 | 0 | 0 | 0 | 0 | 0 | 0 | 0 | 0 |
| Internal collaboration | 1 | 0 | 0 | 1 | 0 | 0 | 0 | 0 | 0 | 0 | 0 | 0 | 0 |
| Continuity of care | 1 | 0 | 0 | 0 | 0 | 0 | 0 | 0 | 0 | 0 | 0 | 1 | 0 |
| Regional differences | 1 | 0 | 1 | 0 | 0 | 0 | 0 | 0 | 0 | 0 | 0 | 0 | 0 |
| Patient management | 1 | 0 | 0 | 0 | 0 | 0 | 0 | 0 | 0 | 0 | 0 | 1 | 0 |
| Clinical observation | 1 | 0 | 0 | 0 | 1 | 0 | 0 | 0 | 0 | 0 | 0 | 0 | 0 |
| Nurse–patient relationship | 1 | 1 | 0 | 0 | 0 | 0 | 0 | 0 | 0 | 0 | 0 | 0 | 0 |
| Helping relationship | 1 | 0 | 0 | 1 | 0 | 0 | 0 | 0 | 0 | 0 | 0 | 0 | 0 |
| Psychological support | 1 | 0 | 0 | 0 | 1 | 0 | 0 | 0 | 0 | 0 | 0 | 0 | 0 |
| Development of clinical competencies | 1 | 0 | 0 | 0 | 1 | 0 | 0 | 0 | 0 | 0 | 0 | 0 | 0 |
| Professional recognition | 1 | 0 | 0 | 0 | 1 | 0 | 0 | 0 | 0 | 0 | 0 | 0 | 0 |
